# Increased Placental Anti-Oxidant Response in Asymptomatic and Symptomatic COVID-19 Third-Trimester Pregnancies

**DOI:** 10.3390/biomedicines10030634

**Published:** 2022-03-09

**Authors:** Alessandro Rolfo, Stefano Cosma, Anna Maria Nuzzo, Chiara Salio, Laura Moretti, Marco Sassoè-Pognetto, Andrea Roberto Carosso, Fulvio Borella, Juan Carlos Cutrin, Chiara Benedetto

**Affiliations:** 1 Department of Surgical Sciences, University of Turin, 10126 Turin, Italy; alessandro.rolfo@unito.it (A.R.); a.nuzzo@unito.it (A.M.N.); l.moretti@unito.it (L.M.); 2 Gynecology and Obstetrics 1, Department of Surgical Sciences, City of Health and Science, University of Turin, 10126 Turin, Italy; stefano.cosma@unito.it (S.C.); andrea88.carosso@gmail.com (A.R.C.); fulvio.borella87@gmail.com (F.B.); 3 Department of Veterinary Sciences, University of Turin, 10095 Grugliasco, Italy; chiara.salio@unito.it; 4 Department of Neuroscience “Rita Levi Montalcini”, University of Turin, 10126 Turin, Italy; marco.sassoe@unito.it; 5 Center of Imaging Molecular, Department of Molecular Biotechnology and Sciences for the Health, University of Turin, 10126 Turin, Italy

**Keywords:** COVID-19, pregnancy, placenta, oxidative stress, mitochondria

## Abstract

Despite Severe Acute Respiratory Syndrome Coronavirus 2 (SARS-CoV-2) -induced Oxidative Stress (OxS) being well documented in different organs, the molecular pathways underlying placental OxS in late-pregnancy women with SARS-CoV-2 infection are poorly understood. Herein, we performed an observational study to determine whether placentae of women testing positive for SARS-CoV-2 during the third trimester of pregnancy showed redox-related alterations involving Catalase (CAT) and Superoxide Dismutase (SOD) antioxidant enzymes as well as placenta morphological anomalies relative to a cohort of healthy pregnant women. Next, we evaluated if placental redox-related alterations and mitochondria pathological changes were correlated with the presence of maternal symptoms. We observed ultrastructural alterations of placental mitochondria accompanied by increased levels of oxidative stress markers Thiobarbituric Acid Reactive Substances (TBARS) and Hypoxia Inducible Factor-1 α (HIF-1α) in SARS-CoV-2 women during the third trimester of pregnancy. Importantly, we found an increase in placental CAT and SOD antioxidant enzymes accompanied by physiological neonatal outcomes. Our findings strongly suggest a placenta-mediated OxS inhibition in response to SARS-CoV-2 infection, thus contrasting the cytotoxic profile caused by Coronavirus Disease 2019 (COVID-19).

## 1. Introduction

A cause–effect relationship between abnormal placental response, maternal COVID-19 severity, and neonatal outcome has not been established to date. Inflammatory changes in SARS-CoV-2-positive placentae are commonly reported. The prevalence of chronic villitis of unknown origin is higher in placentae delivered by COVID-19 patients than in those of healthy controls [1]. At present, the extent to which the vertical transmission of SARS-CoV-2 occurs and the timing of such transmission are unclear. Although SARS-CoV-2 intrapartum transmission is possible [2], low incidence rates of in utero transmission [3,4,5,6] and early and late adverse obstetric outcomes [7,8] suggest that the placenta may play a critical role in modulating maternal response to SARS-CoV-2 infection. The main variable is placental permissibility to SARS-CoV-2 entry mechanisms initiated by spike protein (S) attachment to the angiotensin-converting enzyme 2 (ACE2) receptor [9,10,11]. Studies in murine models demonstrated that maternal hypoxia and oxidative stress (OxS) modulate the expression of SARS-CoV-2 entry machinery [12,13,14] in decidual perivascular and stromal cells, villous cytotrophoblasts, and syncytiotrophoblast cells [15].

In vitro and in vivo studies have revealed the strategy of certain viruses to alter the cellular redox balance in order to survive and to induce OxS, facilitating their replication [16,17]. A defective redox balance in host cells increases viral pathogenesis, resulting in massive cell death [18]. Oxidative imbalance contributes to cell-to-cell viral transmission by modulating innate and adaptive immune responses which exacerbate cytokine/chemokine production and cytokine storms [19] accompanied by overexpression of hypoxia inducible factor 1α (HIF-1α), a key oxygen sensor responsible for cellular adaptation to hypoxia/oxidative stress [20,21].

In experimental animal models, Reactive Oxygen Species (ROS) levels and altered antioxidant defense have been found during SARS-CoV infection [22]. For instance, redox-sensitive transcription factors, such as nuclear factor (NF)-kB, are activated, which induces the transcription of pro-inflammatory Interleukine 1b (IL-1b), IL-6, Tumor Necrosis Factor α (TNF-α), and of adhesion molecules. OxS triggered by SARS-CoV amplifies host response, leading to acute lung injury. SARS-CoV-infected macaques demonstrated lung expression of cytokines and chemokines (e.g., IP-10, MCP-1, IL-6, and IL-8) with a similar pattern as reported in humans [23]. Alterations in enzymatic antioxidant defense mediated by catalase (CAT), superoxide dismutase (SOD), and non-enzymatic antioxidants can be observed in various tissues of patients infected with SARS-CoV-2 [24].

During pregnancy, the high metabolic demand to sustain normal fetal development increases the OxS burden [25]. The placenta is filled with mitochondria with high metabolic activity, making it the main ROS source during pregnancy [26]. Placental ROS play an important role in the regulation of cell signaling in response to a variety of stimuli and insult from perturbations in maternal blood supply to the placenta and inflammation [27]. In addition, the placenta also has a complex antioxidant system (e.g., SOD and CAT enzymes) that usually maintains the action of ROS in balance [28]. When the placental production of ROS overwhelms the endogenous antioxidant defenses, OxS occurs in a variety of maternal, placental, and fetal conditions including gestational diabetes, preeclampsia, and placental insufficiency [26].

SARS-CoV-2-induced OxS regulation is well documented in different organ systems. However, the molecular pathways underlying placental OxS expression during late pregnancy in women with SARS-CoV-2 infection are poorly understood. Moreover, there is limited information about ultrastructural changes occurring in mitochondria during SARS-CoV-2 infection. To fill this gap, we performed an observational study to determine whether the placentae of women testing positive for SARS-CoV-2 during the third trimester of pregnancy showed redox-related alterations involving anti-oxidant CAT and SOD enzymes compared to a cohort of healthy pregnant women. In parallel, we used electron microscopy to investigate mitochondria morphological anomalies, which have been linked to enhanced ROS formation [29,30,31]. Our secondary aim was to determine whether placental redox-related alterations and mitochondria pathological changes were correlated with maternal symptoms of COVID-19 infection.

## 2. Materials and Methods

### 2.1. Study Population and Tissue Collection

Forty-one patients were recruited at Gynecology and Obstetrics 1, Sant Anna University Hospital, City of Health and Science, University of Turin (Turin, Italy). Consecutive full-term pregnant women attending our institution for delivery and testing positive for SARS-CoV-2 nasopharyngeal swab between 1 November and 31 December 2020 were invited to participate in the study (case group). Nasopharyngeal swab samples were taken for reverse transcriptase-polymerase chain reaction (RT-PCR) assay with a dedicated kit (Liferiver Bio-Tech, San Diego, CA, USA) to detect SARS-CoV-2. Two groups were formed: an asymptomatic (a; *n* = 14) and a symptomatic (s; *n* = 15) group. The control group (*n* = 12) was composed of women with a normal-term, healthy singleton pregnancy who showed no signs of maternal, placental, or fetal disease and who had participated in a study conducted before 2018. At that time, none of the patients in either group were vaccinated against COVID-19. The historical data from the control group served to rule out SARS-CoV-2 exposure. The most common indications for cesarean delivery in the control group were fetal malpresentation, previous cesarean section, and maternal request. Four full-thickness tissue biopsies (*n* = 4) from case and control placentae were randomly collected from the intermediate area of the basal plate and snap-frozen immediately after delivery. Next, each single biopsy was processed for both mRNA and protein isolation. Each biopsy was analyzed separately from the others and an average per placenta was obtained and reported. Calcified, necrotic, and seriously damaged areas were excluded from collection. Moreover, placental samples (*n* = 18, six for each clinical group) were preserved in glutaraldehyde combined with formaldehyde for transmission electron-microscopy analyses. Maternal demographics, obstetric, neonatal outcomes, and COVID-19-related maternal symptoms were recorded.

### 2.2. Transmission Electron Microscopy (TEM)

Placental tissue biopsies were fixed by immersion in 1% formaldehyde + 2% glutaraldehyde in phosphate buffer (PB, 0.2 M, pH 7.4) at 4 °C. After washing in PB, they were post-fixed in osmium ferrocyanide for 1 h at 4 °C, dehydrated in graded acetone, and incubated in acetone/Spurr resin (1:1:30 min; 1:2:30 min) and Spurr resin overnight at room temperature. Finally, samples were embedded in Spurr resin in 0.5 mL Eppendorf tubes (24 h, 70 °C). Ultrathin sections were cut with an ultramicrotome (EM UC6, Leica Microsystems, Wetzlar, Germany), collected on uncoated nickel grids (200 mesh), and counterstained for 30 s with Uranyl Less EM Stain and for 30 s with Lead citrate (Electron Microscopy Sciences, Hatfield, PA, USA). Placental sections were observed with a JEM-1400 Flash transmission electron microscope (JEOL, Tokyo, Japan), and images acquired with a high-sensitivity sCMOS camera. A total of eighteen placentae (six from control pregnancies and twelve from COVID-19-infected pregnant women) were evaluated. The COVID-19 group comprised placentae from six a-COVID-19 women and six s-COVID-19 women.

### 2.3. Lipid Peroxidation Measurement

Since ROS are highly reactive and have a very short half-life, direct detection with accuracy and precision in tissue and body fluids is often unfeasible [32]. Counterwise, peroxyl radicals and hydrogen peroxide are relatively stable molecules (with half-lives of seconds to minutes). Therefore, a promising alternative approach to measure oxidative stress in clinical samples is indirect measurement of ROS by examining the oxidative damage these radicals cause to the cell lipids, proteins, and nucleic acids [33]. For the present study, the degree of placenta lipid peroxidation of the plasma membranes was estimated by measuring the Thiobarbituric Acid-Reactive Substances (TBARS) by means of a TBARS Assay Kit (Cayman chemical, Ann Arbor, MI, USA). Absorbance was measured at 535 nM on an ELISA SR 400 microplate reader and the TBARS values were calculated using a Malondialdehyde (MDA) standard curve, prepared by acid hydrolysis of 1,1,3,3-tetramethoxypropane. The values are expressed as MDA μM.

### 2.4. RNA Isolation and Real-Time PCR

Total RNA was isolated from frozen placental biopsies using TRI^®^ reagent (Sigma-Aldrich, Milan, Italy) according to the manufacturer’s instructions and then treated with DNase I to remove genomic DNA contamination. Three micrograms of total RNA was reverse-transcribed using a random-hexamer approach (Fermentas Europe, St. Leon-Rot, Germany) and a RevertAid H Minus First Strand cDNA synthesis kit (Fermentas, Cat. No k1632, Leon-Rot, Germany). qRT-PCR reactions were run on a StepOne™ real-time PCR system instrument (Applied Biosystems, Waltham, MA, USA). Gene expression levels of hypoxia-inducible factors 1 α (HIF-1α), CAT, and SOD1 were determined by real-time PCR using specific TaqMan primers and probes following the manufacturer’s protocol (Life Technologies, Carlsbad, CA, USA, Cat. No 4331182). TaqMan primers and probes for ribosomal 18S, HIF-1α, CAT, and SOD1 were purchased from Applied Biosystems as TaqMan gene expression assays. For relative quantification, PCR signals were compared between the groups after normalization using ribosomal 18S RNA expression as an internal reference (Life Technologies, Carlsbad, CA, USA, Cat. No 4333760F). Relative expression and fold change were calculated according to Livak and Schmittgen [34].

### 2.5. Assessment of SOD and CAT Enzymatic Activities

CAT and SOD enzyme activity in the placental biopsies was determined using commercially available kits (Cayman Chemical, Ann Arbor, MI, USA) and following the manufacturer’s instructions. Briefly, CAT activity was determined by measuring catalase peroxidative function based on the reaction between CAT and methanol in the presence of optimum concentration of hydrogen peroxide. Formaldehyde was measured spectrophotometrically at 540 nm using 4 amino-3-hydrazino-5-mercapto-1,2,4-triazole. Results are expressed in nmol/min/mL. Total SOD activity was measured by reduction of cytochrome C by superoxide (O_2_•−) radicals monitored spectrophotometrically at 450 nm using the xanthine-xanthine oxidase system. Results are expressed in U/mL.

### 2.6. Statistical Analysis

Data are presented as median ± SEM (standard error of the median) and the Kruskal–Wallis non-parametric test was used since data did not show the same distribution. If a significant difference was found between groups, the Mann–Whitney U-test with Bonferroni’s correction was performed. Categorical variables are presented as frequency (percentages); the chi-square test was performed for comparison between groups. Statistical analysis was carried out using SPSS Version 27 statistical software (IBM Corp. Released 2020. IBM SPSS Statistics for Windows, Version 27.0. Armonk, NY, USA: IBM Corp.). Significance was accepted at *p* < 0.05.

## 3. Results

### 3.1. Clinical Features of Study Population

Table 1 presents the clinical features of the study population. The case and the control groups were comparable for maternal age and gestational age at delivery, and percentage of nulliparous women, cesarean section, and female and male neonates. A total of 29 (70%) pregnant women tested positive for SARS-CoV-2 infection, of which 15 (51%) were categorized as symptomatic (s-COVID-19) according to previously published criteria [35] and 14 (48%) as asymptomatic (a-COVID-19). The control (CTRL) group was composed of 12 women. As expected, the incidence of overweight was higher among the women testing positive for COVID-19; there was no statistically significant difference between the a-COVID-19 and s-COVID-19 groups. No stillbirths were recorded and no neonatal respiratory support was required within 24 h of birth in the SARS-CoV-2-positive women during pregnancy. There were no differences in placental or birth weight or percentage of female and male neonates between groups. However, a significantly higher percentage of female neonates was reported within the a-COVID-19 and s-COVID-19 groups (71.4 and 60%, respectively; *p* < 0.0001). In the CTRL group, we reported a higher percentage of male neonates (58.3%; *p* = 0.001). Finally, an increased percentage of abnormal cardiotocography (CTG) was noted in the a-COVID-19 and s-COVID-19 groups (21.4% and 26.7%, respectively) compared to the CTRL group, even though no significant differences were reported between the a-COVID-19 and s-COVID-19 groups.

### 3.2. Placenta Ultrastructural Morphology in a-COVID-19, s-COVID-19, and CTRL Pregnant Women

**Figure 1:**
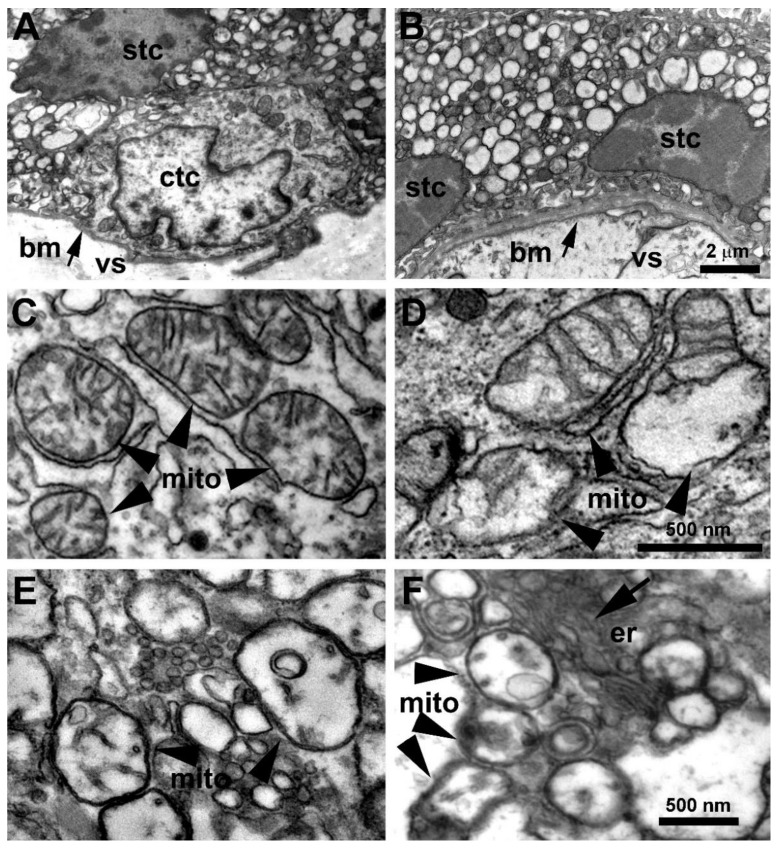
Effects of SARS-CoV-2 infection on placenta ultrastructure. (**A**,**B**) Representative images of a placenta from a control woman (**A**) and a SARS-CoV-2-infected woman (**B**) showing cells of syncytiotrophoblast (stc) and cytotrophoblast (ctc); arrows indicate the basal membrane (bm) that separates the trophoblast layer from the villous stroma (vs). Note the large amount of vacuolar structures infiltrating the cytoplasm of trophoblast cells. (**C**) Higher magnification showing mitochondria (mito) in a syncytiotrophoblast cell from a control placenta. Note the normal aspect of cristae. (**D**) Mitochondria in a trophoblast cell from a placenta of a woman with COVID-19. Note the presence of enlargements with noticeable reduction in cristae. The remaining cristae appear somewhat expanded. (**E**,**F**) Severely altered mitochondria in the placentae of women with COVID-19. Note the electro-lucent matrix, the strong reduction in cristae, and the presence of abnormal membranous inclusions. er: endoplasmic reticulum.

We used transmission electron microscopy to assess ultrastructural alterations in the placentae of women with COVID-19. Figure 1A,B illustrate the general appearance of trophoblast cells in the placenta of control (A) and SARS-CoV-2-infected (B) women. In both groups, cells were filled with vacuolar structures of variable size interspersed with other cellular organelles. Mitochondria are shown at higher magnification in panels C–F. While in the placentae from CTRL group the mitochondria had a normal appearance (Figure 1C), ultrastructural alterations were observed in placentae of both a-COVID-19 and s-COVID-19 patients (Figure 1D–F). Specifically, these organelles frequently displayed swellings with a reduction in the number of cristae (Figure 1D). Some of the mitochondria presented severe alterations consisting in matrix rarefaction, cristae loss, and the formation of abnormal membranous structures (Figure 1E,F). These alterations were invariably observed in all placentae from SARS-CoV-2-infected women but not in control placentae. It is of note that viral particles were not detected unambiguously in these samples.

### 3.3. Assessment of Placental Oxidative Stress Markers

**Figure 2:**
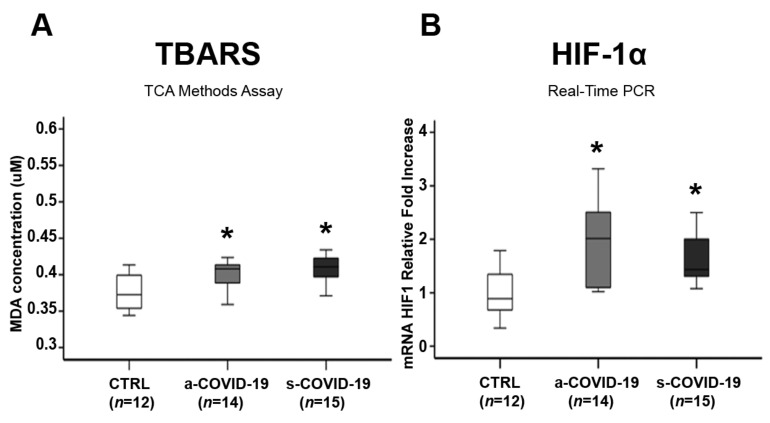
Effects of SARS-CoV-2 infection on oxidative stress markers in the placenta from the a-COVID-19, s-COVID-19, and CTRL groups. (**A**) TBARS and (**B**) HIF-1α expression in the placenta from the CTRL, a-COVID-19, and s-COVID-19 groups. Statistical significance was set at *p* < 0.05. * *p* < 0.05 versus CTRL. The Kruskal–Wallis test and Mann–Whitney U-test with a Bonferroni correction were used.

There was a significant increase in TBARS levels as a lipid peroxidation biomarker in the a-COVID-19 (*p* = 0.018, 1.1-fold increase) and the s-COVID-19 group (*p* = 0.003, 1.1-fold increase) placentae compared to the CTRL group (Figure 2A), which indicated placental OxS onset after SARS-CoV-2 infection. No significant differences were reported between the a-COVID-19 and s-COVID-19 groups (*p* > 0.05). Placental OxS in the COVID-19-positive women was detected by hypoxia-inducible factors 1 α (HIF-1 α), a key transcription factor that regulates cellular response to hypoxia and plays a critical role in ROS production and OxS onset. We found significant HIF-1 α overexpression in the placentae of the a-COVID-19 (*p* = 0.004, 1.95-fold increase) and the s-COVID-19 (*p* = 0.028, 1.87-fold Increase) groups compared to the CTRL group (Figure 2B). However, no significant differences were reported between the a-COVID-19 and s-COVID-19 groups (*p* > 0.05).

### 3.4. Assessment of Placental Antioxidant Defense Markers

**Figure 3:**
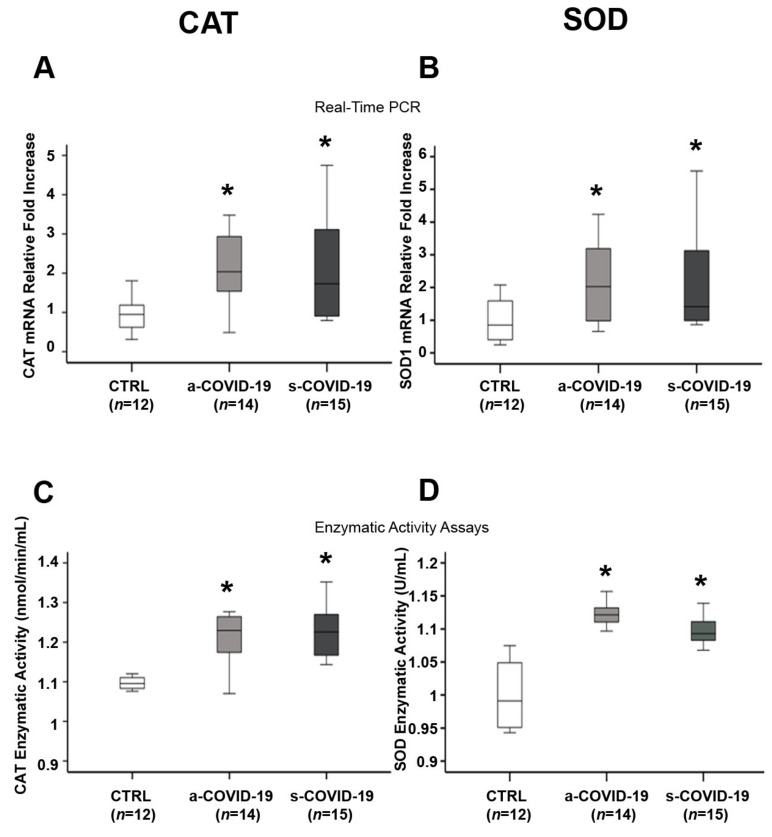
Effects of SARS-CoV-2 infection on antioxidant defense markers in the placenta from the a-COVID-19, s-COVID-19, and CTRL groups. (**A**) mRNA expression and (**C**) enzymatic activities of CAT in the placentae from the CTRL, a-COVID-19, and s-COVID-19 groups; (**B**) mRNA expression and (**D**) enzymatic activity of SOD in the placentae from the CTRL, a-COVID-19, and s-COVID-19 groups. Statistical significance was set at *p* < 0.05. * *p* < 0.05 versus CTRL. The Kruskal–Wallis test and Mann–Whitney U-test with Bonferroni correction were used.

Endogenous defense against an abundance of pro-oxidant agents involves the overall action of antioxidant enzymes to detoxify the free radicals and avert tissue damage. SOD1 catalyzes the conversion of the O_2_●− radical to H_2_O_2_, then cytosolic CAT transforms H_2_O_2_ to water. We noted significantly higher CAT mRNA levels in the placenta of the a-COVID-19 (*p* = 0.006, 2.16-fold increase) and s-COVID-19 (*p* = 0.026, 2.19-fold increase) groups compared to the CTRL group (Figure 3A). However, no significant differences were reported between the a-COVID-19 and s-COVID-19 groups (*p* > 0.05). CAT enzymatic activity was significantly increased in the a-COVID-19 (*p* = 0.04, 1.1-fold increase) and s-COVID-19 (*p* = 0.013, 1.11-fold increase) groups compared to the CTRL group (Figure 3C), while no significant differences were reported between the a-COVID-19 and s-COVID-19 groups (*p* > 0.05). CAT overexpression was accompanied by a significant increase in SOD mRNA levels in the placentae of the a-COVID-19 (*p* = 0.014, 2.12-fold increase) and s-COVID-19 (*p* = 0.041, 2.36-fold increase) groups compared to the CTRL group (Figure 3B). SOD gene overexpression was observed, with a significant increase in SOD enzymatic activity in the a-COVID-19 (*p* = 0.004, 1.12-fold increase) and s-COVID-19 (*p* = 0.011, 1.1-fold increase) groups compared to the CTRL group (Figure 3D). No significant differences in SOD gene expression levels and enzymatic activities were reported between the a-COVID-19 and s-COVID-19 groups (*p* > 0.05).

### 3.5. Comparisons of Placental Oxidative Stress and Antioxidant Defense Markers in SARS-CoV-2-Infected Women with and without Pregnancy-Related Comorbidities

**Figure 4:**
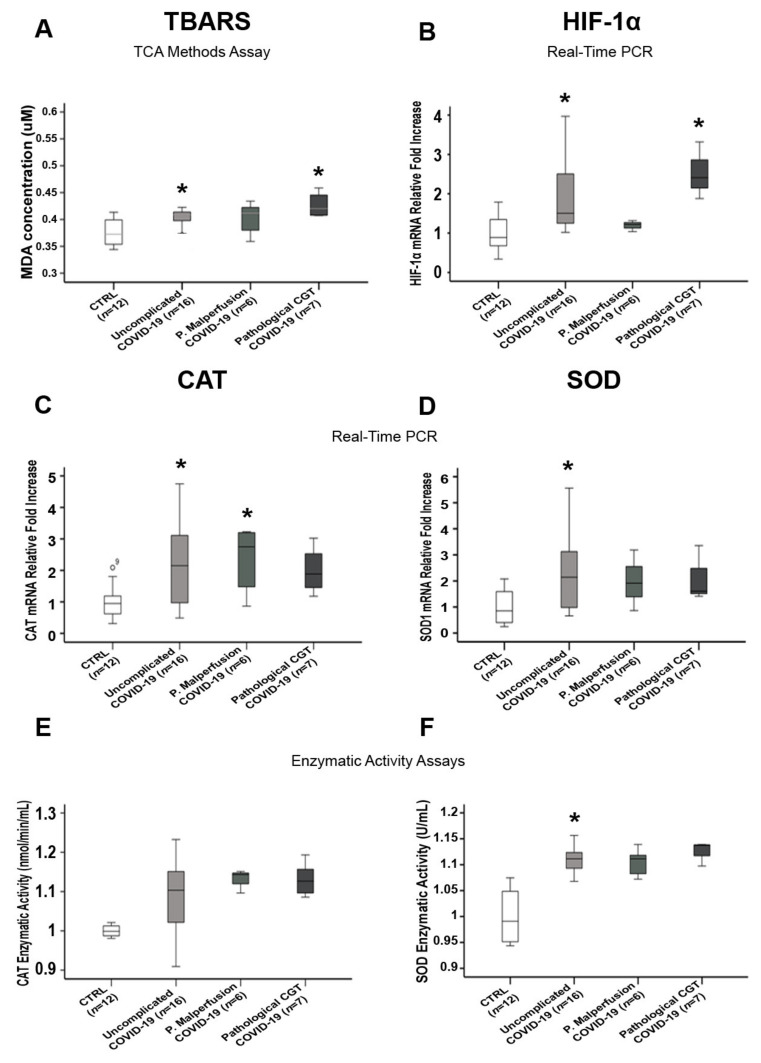
TBARS, HIF-1α, CAT, and SOD expression in the placentae of COVID-19-positive women with and without pregnancy-related comorbidities. (**A**) TBARS and (**B**) HIF-1α placental expression in CTRL, uncomplicated COVID-19, Placental malperfusion (P. Malperfusion) COVID-19, and Pathological CTG COVID-19 groups; (**C**) mRNA expression and (**E**) enzymatic activity of CAT in the placentae of the CTRL, uncomplicated COVID-19, Placental malperfusion COVID-19, and Pathological CTG COVID-19 groups; (**D**) mRNA expression and (**F**) enzymatic activity of SOD in the placentae of the CTRL, uncomplicated COVID-19, Placental malperfusion COVID-19, and Pathological CTG COVID-19 groups. Statistical significance was set at *p* < 0.05. * *p* < 0.05 versus CTRL. The Kruskal–Wallis test and Mann–Whitney U-test with a Bonferroni correction were used.

We compared TBARS, HIF-1α, CAT, and SOD levels between COVID-19-positive women with and without complications during pregnancy and the CTRL group in order to rule out the potential contribution of pregnancy-related comorbidity (gestational hypertension, intrauterine growth restriction, preeclampsia) or abnormal CTG-to-OxS markers and anti-oxidant overexpression. We found a significant increase in the OxS markers TBARS (*p* = 0.031) and HIF-1 α (*p* = 0.01) (Figure 4A,B) and anti-oxidant CAT (gene, *p* = 0.039; enzymatic activity, *p* > 0.05) and SOD (gene, *p* = 0.016; enzymatic activity, *p* = 0.006) (Figure 4C–F) in the COVID-19 women compared to the CTRL group. There were no significant differences between the SARS-CoV-2-positive women who went through pregnancy without comorbidities and those with a pregnancy-related comorbidity (*p* > 0.05, Figure 4).

## 4. Discussion

To our best knowledge, this is the first report of alterations of placental mitochondria associated with increased levels of oxidative stress markers TBARS and HIF-1α in women with SARS-CoV-2 infection during the third trimester of pregnancy. Our data confirm the known COVID-19 pro-oxidant and cytotoxic profile of the placenta. Importantly, our results were associated with an increase in placental SOD and CAT anti-oxidant enzymatic activities accompanied by physiological neonatal outcomes providing clues for a compensatory adaptation of the placenta to maintain its physiological abilities and to protect fetal growth. Our observational findings strongly suggest placenta-mediated OxS inhibition in response to SARS-CoV-2 infection, thus contrasting the cytotoxic profile caused by COVID-19.

In line with Gao and colleagues [36], we found no evidence of unfavorable obstetric and neonatal outcomes in women infected by SARS-CoV-2 during the third trimester. The clinical manifestations of SARS-CoV-2 in the pregnant women were similar to those seen in the general population. As reported elsewhere [37], the most common symptom in the symptomatic COVID-19 group was fever. Previous studies [9,37,38,39] reported that SARS-CoV-2 infection predominantly affects pregnant women over the age of 30 years. Maternal COVID-19 has also been linked with iatrogenic preterm birth due to maternal indications, but the overall rates of spontaneous preterm births are not high and the rates of stillbirths and neonatal deaths do not seem to be any higher than the background rates [40,41,42,43]. This suggests that placental trophoblasts may be less susceptible to SARS-CoV-2 infection during the third trimester of pregnancy because of decreased ACE2 expression [37]. Similar to previous reports [44,45], we recorded no cases of vertical transmission of SARS-CoV-2. The most frequent comorbidities that we recorded were gestational overweight, gestational hypertension, and hematological disorders. These observations are shared by other reports [37,39,46].

The strong perturbation of mitochondria morphology that we observed in COVID-19 placentae was previously described in lung epithelial cells [47], and it is often associated with functional alterations comprising inhibited mitochondrial biogenesis, loss of mitochondrial membrane potential (MMP or ΔΨm), and inhibition of oxidative phosphorylation [47,48]. In a recent publication, it was suggested that SARS-CoV-2 RNA transcripts and open-reading frames (ORFs) such as ORF 9 localize in mitochondria and regulate mitochondrial function [49]. Hijacked mitochondrial functions constitute a favorable condition to increase the steady-state levels of reactive oxygen species (ROS) production [48,50]. These events are recognized as major COVID-19 pathogenic mechanisms suggestive of an altered OxS regulation triggered by SARS-CoV-2 that we confirmed with placental TBARS and HIF-1α overexpression.

As for other beta-coronaviruses’s family members, SARS-CoV-2 replicates in the cytoplasm. Nevertheless, we could not unambiguously identify viral particles in placentae from COVID-19 pregnant women. Drastic cytoplasm vacuolization that we reported in SARS-CoV-2 trophoblast cells was previously described in an in vitro model of human conducting airway epithelium and Madin–Darby bovine kidney cells for SARS-CoV [51,52] leading to the hypothesis that vacuoles, in terms of early and late endosomes, could have a key role in the virus assembly process [53]. Accordingly, it was recently proposed that the downregulation of Rab7 small GTPase protein found in placentae from COVID-19 pregnant women could result in retention of the virus in the early endosomes or trapping within late endosomes and MVB, mediating physiological placental blockade of SARS-CoV-2 in pregnancy and consequently its vertical transmission. This hypothesis would help to explain why the presence of SARS-CoV-2 material in the placenta constitutes a rare event and not the rule [54].

Ultrastructural mitochondria alterations are likely correlated with the elevated oxidative stress documented herein by TBARS and HIF-1α overexpression in COVID-19 placentae. This association was previously described in patients with COVID-19 [17,55,56,57,58,59]. OxS contributes to SARS-CoV-2 pathogenesis and severity by inducing inflammation, loss of immune function, and by increasing viral replication which may result from activation of the nuclear factor kappa B (NF-κB) pathway [58,60]. Moreover, RNA viruses promote changes in the body’s antioxidant defense system and affect enzymes such as SOD and CAT [61,62]. Serum CAT and SOD levels were found to be lower in COVID-19 patients than in controls [63]. In contrast, we demonstrated a statistically significant increase in CAT and SOD enzymatic activity in the placenta from COVID-19 women compared to the controls. A plausible explanation is that the placenta mounts a defense mechanism to fight SARS-CoV-2-induced OxS and to ensure physiological fetal growth and development, as we found in the present cohort.

Furthermore, there is ample evidence that the placenta can counteract adverse conditions (e.g., maternal nutritional challenges, glucocorticoid overexposure, hypoxia) [64,65,66]. In detail, the antioxidant activity by the placenta in response to oxidative stress was demonstrated in preeclamptic pregnancies that reached term delivery by making multiple adaptations in mitochondrial function and related processes that were only minimally observed in preeclamptic pregnancies that delivered pre-term [67].

Our study is limited by having included patients who were infected with the first form of SARS-CoV-2 and by the fact that, today, the spread of COVID-19 vaccines has reached acceptable levels in many countries. The immune and placental response in these women may be different. However, there is a high proportion of women who preferred not to undergo vaccination and there are many developing countries where vaccines are not yet widely available. Our observations are therefore of interest in understanding the placental effects of infection in unvaccinated patients.

Researchers interested in studying the placental effects of SARS-CoV-2 will face the possibility that their data will be changed by the effects of immunization. Our experimental model offers a “clean” image of this confounder: the analysis of our samples does not present the risk of presenting different characteristics due to the effect of immunization or infection with subsequent variants (for example Omicron). For these reasons, we consider our results to be original and difficult to be reproduced in the future.

## 5. Conclusions

In conclusion, we observed that SARS-CoV-2 infection during the third trimester of pregnancy induced placental mitochondrial alterations, terminal end products of lipid peroxidation, and an antioxidant adaptation most likely to minimize the detrimental effects of COVID-19-induced OxS on fetal development. Our data suggest that the redox-regulated intracellular pathways triggered by SARS-CoV-2 infection may offer a novel therapeutic target for COVID-19 during pregnancy.

## Figures and Tables

**Table 1:** Clinical features of COVID-19-positive and CTRL groups. Values are expressed as median ± SEM and percentage. Significant differences (*p* < 0.05): ^ differences indicating a significant effect compared with CTRL; * differences indicating a significant effect compared with a-COVID-19. The Kruskal–Wallis test, Mann–Whitney U-test with a Bonferroni correction, and chi-square tests were used.

	CTRL (*n* = 12)	a-COVID-19 (*n* = 14)	s-COVID-19 (*n* = 15)	*p* Value
Nulliparae (%)	14.3 (*n* = 2)	35.7 (*n* = 5)	53.3 (*n* = 8)	*p* > 0.05
Maternal age at delivery (years)	35.1 ± 0.8	32 ± 1	33.1 ± 0.7	*p* > 0.05
Gestational age at delivery (weeks)	39.8 ± 0.2	38.8 ± 0.8	37.8 ± 0.5	*p* > 0.05
Pre-Pregnancy comorbidity:				
Asthma (%) Diabetes (%) Tobacco (%) Chronic Hypertension (%) Gestational Overweight (%) Hematological Disease (%)	0 0 0 0 0 0	0 21.4 (*n* = 3) 0 0 35.7 ^^^ (*n* = 5) 14.3 (*n* = 2)	0 6.7 (*n* = 1) 6.7 (*n* = 1) 0 13.3 (*n* = 2) 6.7 (*n* = 1)	*p* > 0.05 *p* > 0.05 *p* > 0.05 *p* > 0.05 ^^^ *p* = 0.021 *p* > 0.05
Pregnancy complications:				
Gestational Hypertension (%) IUGR (%) HELLP (%) PE (%)	0 0 0 0	21.4 (*n* = 3) 7.1 (*n* = 1) 0 0	6.7 (*n* = 1) 0 0 6.7 (*n* = 1)	*p* > 0.05 *p* > 0.05 *p* > 0.05 *p* > 0.05
SARS-CoV-2 symptoms:				
Dyspnea (%) Fever (%) Anosmia/Ageusia/Asthenia (%) Cough (%) Rhinitis (%)	0 0 0 0 0	0 0 0 0 0	40 (n = 6) 86.7 ^^^^,^* (*n* = 13) 66.7 ^^^^,^* (*n* = 10) 66.7 ^^^^,^* (*n* = 10) 13.3 (*n* = 2)	*p* > 0.05 ^^^^,^* *p* = 0.001 ^^^^,^* *p* = 0.001 ^^^^,^* *p* = 0.001 *p* > 0.05
Obstetrics & Neonatal outcomes				
Pathological Doppler (%) Pathological CTG (%) Cesarean section (%) Birth weight (g) Placental weight (g) Positive neonatal swab (%) APGAR < 7 at 5 min (%) Female fetus (%) Male fetus (%)	0 0 41.7 (*n* = 5) 3358 ± 121.2 571 ± 22.2 0 0 41.7 (*n* = 5) 58.3 (*n* = 7)	0 21.4 (*n* = 3) 35.7 (*n* = 5) 3306.4 ± 191.7 604.1 ± 46.6 0 7.1 (*n* = 1) 71.4 (*n* = 10) 28.6 (*n* = 4)	6.7 (*n* = 1) 26.7 (*n* = 4) 60 (*n* = 9) 2993 ± 171.7 554.3 ± 40.3 0 6.7 (*n* = 1) 60 (*n* = 9) 40 (*n* = 6)	*p* > 0.05 *p* > 0.05 *p* > 0.05 *p* > 0.05 *p* > 0.05 *p* > 0.05 *p* > 0.05 *p* > 0.05 *p* > 0.05

## Data Availability

The raw data supporting the conclusions of this article will be made available by the authors without undue reservation.
